# Supplementary material for: Maternal age alters offspring lifespan, fitness, and lifespan extension under caloric restriction
Source: Sci Rep. 2019 Feb 28;9:3138. doi: 10.1038/s41598-019-40011-z (PMC6395700; doi:10.1038/s41598-019-40011-z)
Supplement: Supplementary file 1 — Supplementary Information: Maternal age alters offspring lifespan, fitness, and lifespan extension under caloric restriction [file 41598_2019_40011_MOESM1_ESM.pdf]

Supplementary Information: Maternal age alters offspring lifespan, fitness and lifespan extension under caloric restriction

Martha J. Bock[1,2‡], George C. Jarvis[1,3‡], Emily L. Corey[1], Emily E. Stone[1], and Kristin E. Gribble[1*]

[1]Marine Biological Laboratory, Woods Hole, MA, USA 02543
[2]Mayo Clinic, Rochester, MN, USA 55905
[3]California State University, Northridge, Northridge, CA, USA 91330
[‡]These authors contributed equally to this work

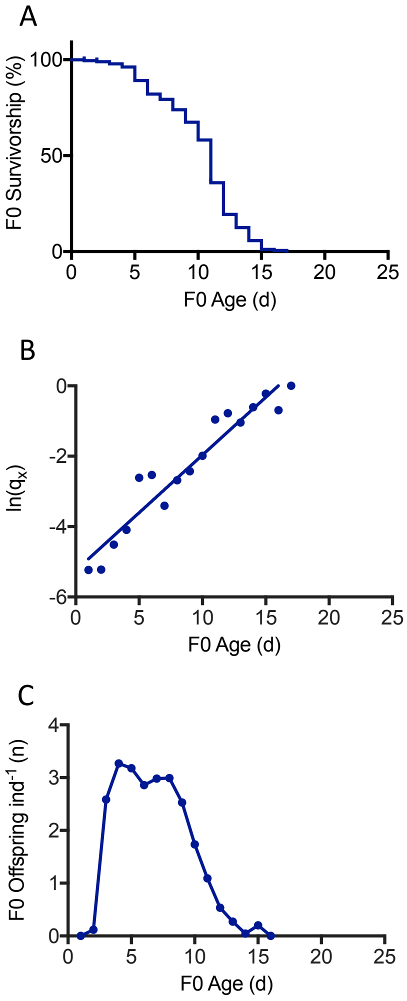

Supplementary Fig. 1. Maternal (F0) survivorship **(A)**, hazard rate **(B)**, and daily reproduction rate **(C)**. n = 187.

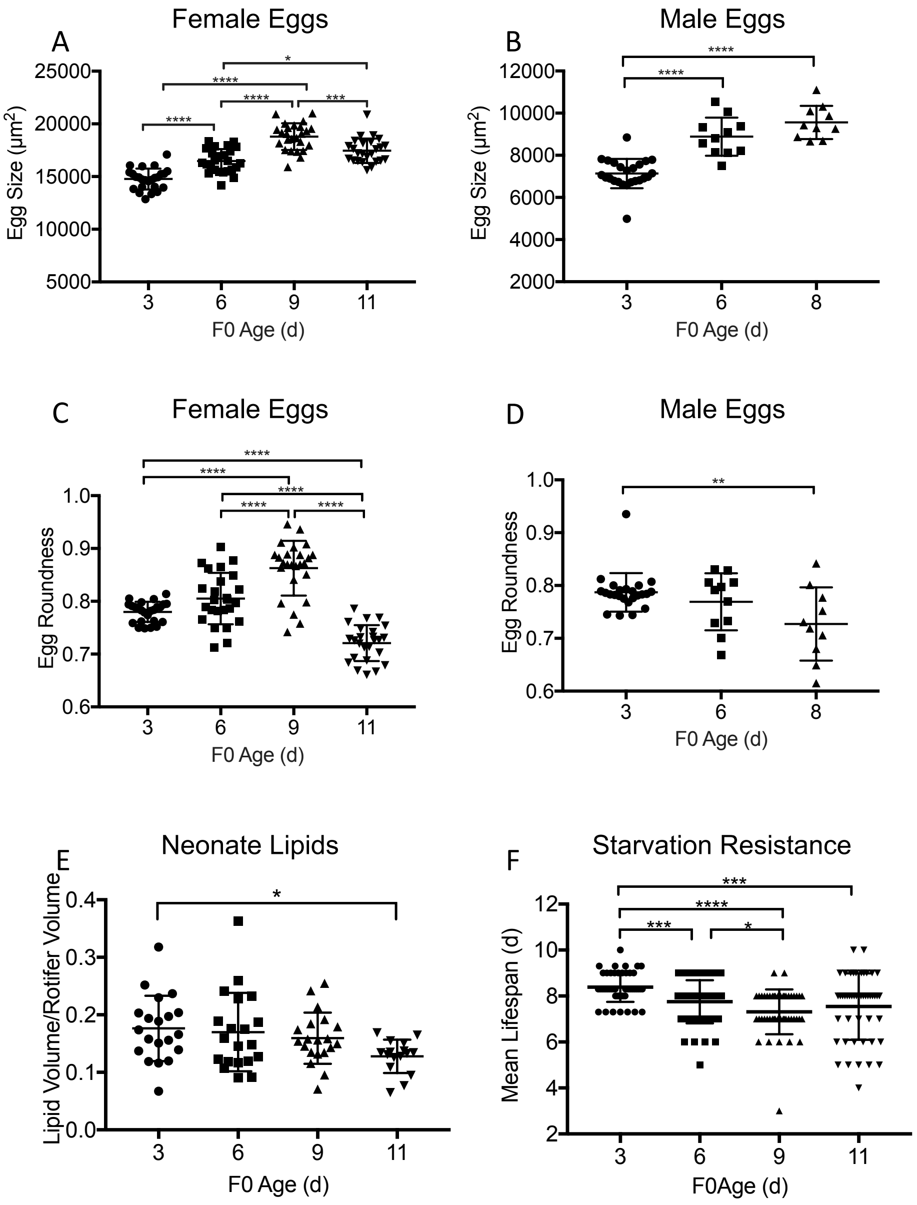

Supplementary Fig. 2. Maternal investment in offspring. Amictic egg (hatch into females, n = 20) and mictic egg (hatch into males, n = 10) size (**A**, and **B**, respectively) and shape (**C** and **D**) from F0 mothers of different ages. All multiple comparisons between every maternal age were significantly different for egg size and shape except for maternal age 3 d versus 6 d for both amictic and mictic egg roundness (one-way ANOVA with Tukey's test for multiple comparisons). Neonate lipid content **(E)** was significantly different only between maternal ages of 3 d and 11 d ($p = 0.04$; one-way ANOVA with Holm-Sidak's test for multiple comparisons, n = 20). Offspring starvation resistance **(F)** was significantly different among all maternal ages except 6 d and 9 d versus 11 d (n = 48). Significance indicated as * ($p < 0.05$), ** ($p < 0.01$), *** ($p < 0.001$), or **** ($p < 0.0001$).

		LIFESPAN					GOMPERTZ					
**Maternal Age Cohort**	**Diet**	**Median Lifespan (d)**	**Percent Change from AL**	**Median Diff than AL? (p)**	**Median Diff than $F1_3$? (p)**	**Maximum lifespan (d; 95%)**	**α (intercept)**	**α Diff than AL? (p)**	**β (slope)**	**β Diff than AL? (p)**	**R^2**	**n**
**F0**	**AL**	11					-5.247 ± 0.2523		0.3281 ± 0.02462		0.922	187
	**AL**	14				16.4	-6.995 ± 0.5566		0.3959 ± 0.04306		0.894	71
**$F1_3$**	**CCR**	16	14.3	**<0.0001**		19.35	-5.714 ± 0.3134	‡	0.2677 ± 0.04306	**0.0109**	0.921	72
	**IF**	17	21.4	**<0.0001**		21.35	-5.478 ± 0.488	‡	0.2251 ± 0.04306	**0.0047**	0.815	72
	**AL**	12			**<0.0001**	16	-6.169 ± 0.7776		0.3715 ± 0.06794		0.769	72
**$F1_5$**	**CCR**	15	**25.0**	**<0.0001**	0.9490	20	-5.727 ± 0.3153	**0.0038**	0.266 ± 0.0232	0.101	0.910	71
	**IF**	13	**8.3**	**0.0004**	**0.0011**	20	-4.508 ± 0.5135	‡	0.1855 ± 0.03497	**0.0188**	0.684	71
	**AL**	12			**<0.0001**	16	-5.395 ± 0.5705		0.3151 ± 0.05162		0.788	71
**$F1_7$**	**CCR**	14	16.7	**<0.0001**	0.1999	21	-5.422 ± 0.3754	**0.0056**	0.2545 ± 0.02842	0.3001	0.851	69
	**IF**	18	**50.0**	**<0.0001**	**0.0183**	24	-5.784 ± 0.4458	**<0.0001**	0.2192 ± 0.02771	0.1021	0.817	72
	**AL**	11			**<0.0001**	16	-4.179 ± 0.4649		0.229 ± 0.04152		0.717	72
**$F1_9$**	**CCR**	13	18.2	**0.0003**	**0.0151**	19.7	-4.037 ± 0.3194	**0.016**	0.1653 ± 0.02428	0.1776	0.768	72
	**IF**	14	27.3	**<0.0001**	**0.0001**	20	-4.804 ± 0.4156	**0.0016**	0.211 ± 0.02946	0.7222	0.786	72

Supplementary Table 1. Changes in lifespan and mortality rate in maternal females (F0) and in offspring from 3, 5, 7, and 9-d old mothers ($F1_3$, $F1_5$, $F1_7$, and $F1_9$, respectively) under *ad libitum* (AL; 6 x 10^5 cells ml^{-1} *Tetraselmis suecica*), chronic caloric restriction (CCR; 6 x 10^4 cells ml^{-1} *T. suecica*, a 90% reduction in food relative to AL), or intermittent fasting (IF; alternate day AL and starvation) diets. Significant differences are shown in bold. ‡ Because slopes differ so much, it is not possible to test whether the intercepts differ significantly. Column 4 shows where the percent change from AL is significantly different from that for $F1_3$ in bold (z test for two population proportions, $p < 0.05$).

	Diet	Net Repro Rate (Ro)	Diff than AL? (p)	Diff than $F1_3$? (p)	Max Daily Repro	Diff than AL? (p)	Diff than $F1_3$? (p)	Non-viable eggs (n)	Diff than AL? (p)	Diff than $F1_3$? (p)
	**AL**	27.30 ± 0.77			3.60 ± 0.12			1.15 ± 0.18		
**$F1_3$**	**CCR**	26.56 ± 0.85	**<0.0001**		3.14 ± 0.09	**0.0010**		0.79 ± 0.13	0.5221	
	**IF**	20.5 ± 0.59	**<0.0001**		2.31 ± 0.07	**<0.0001**		0.43 ± 0.09	0.0773	
	**AL**	24.22 ± 0.86		0.1145	3.73 ± 0.11		0.8088	2.42 ± 0.34		**0.0014**
**$F1_5$**	**CCR**	23.68 ± 0.97	**<0.0001**	0.1586	3.13 ± 0.10	**<0.0001**	0.9609	1.30 ± 0.22	**0.0048**	0.4502
	**IF**	15.29 ± 0.86	**<0.0001**	**0.0016**	2.38 ± 0.10	**<0.0001**	0.6403	1.16 ± 0.29	**0.0019**	0.1709
	**AL**	21.56 ± 1.22		**0.0001**	3.49 ± 0.19		0.8088	1.75 ± 0.29		0.2913
**$F1_7$**	**CCR**	21.11 ± 1.178	**0.0074**	**0.0003**	2.75 ± 0.16	**0.0018**	**0.0017**	1.25 ± 0.22	0.3057	0.5106
	**IF**	17.52 ± 0.66	**0.0205**	0.1159	2.13 ± 0.11	**<0.0001**	0.5060	0.68 ± 0.15	**0.0042**	0.883
	**AL**	14.82 ± 1.25		**<0.0001**	3.06 ± 0.22		0.0618	2.17 ± 0.47		**0.017**
**$F1_9$**	**CCR**	17.20 ± 1.26	0.1998	**<0.0001**	2.53 ± 0.14	0.0529	**0.0011**	0.97 ± 0.16	**0.0018**	0.9535
	**IF**	14.20 ± 0.95	0.9023	**<0.0001**	2.49 ± 0.13	0.0529	0.5060	1.09 ± 0.24	**0.0088**	0.2503

Supplementary Table 2. Changes in reproduction in offspring from 3, 5, 7, and 9-d old mothers ($F1_3$, $F1_5$, $F1_7$, and $F1_9$, respectively) under *ad libitum* (AL; 6 x 10^5 cells ml^{-1} *Tetraselmis suecica*), chronic caloric restriction (CCR; 6 x 10^4 cells ml^{-1} *T. suecica*, a 90% reduction in food relative to AL), or intermittent fasting (IF; alternate day AL and starvation) diets. Significant differences ($p < 0.05$) are shown in bold.

	Diet	Pre-Repro Period (d)	Diff than AL? (p)	Diff than $F1_3$? (p)	Repro Period (d)	Diff than AL? (p)	Diff than $F1_3$? (p)	Post-Repro Period (d)	Diff than AL? (p)	Diff than $F1_3$? (p)
	**AL**	1.28 ± 0.05			9.69 ± 0.22			2.51 ± 0.17		
**$F1_3$**	**CCR**	1.19 ± 0.05	0.7016		12.07 ± 0.40	**0.0003**		1.86 ± 0.18	**0.0001**	
	**IF**	1.26 ± 0.06	0.6308		12.66 ± 0.38	**0.0019**		2.44 ± 0.19	**0.0231**	
	**AL**	1.00 ± 0.0		0.9680	8.56 ± 0.23		0.3610	1.92 ± 0.16		0.6006
**$F1_5$**	**CCR**	1.03 ± 0.03	0.9993	0.9995	10.43 ± 0.37	0.1913	**0.0199**	3.19 ± 0.32	0.1787	**0.0049**
	**IF**	1.30 ± 0.19	0.6206	0.5925	9.17 ± 0.48	0.1946	**0.0347**	2.68 ± 0.38	0.7035	0.3462
	**AL**	1.03 ± 0.03		0.9999	7.39 ± 0.32		**0.0007**	2.79 ± 0.24		**0.0062**
**$F1_7$**	**CCR**	1.11 ± 0.04	0.8261	0.9959	9.87 ± 0.46	0.6690	**<0.0001**	3.92 ± 0.38	0.9438	**<0.0001**
	**IF**	1.41 ± 0.13	0.9819	0.9819	12.49 ± 0.43	**0.0114**	**0.0258**	3.58 ± 0.26	0.0886	0.0570
	**AL**	1.19 ± 0.16		0.0253	6.37 ± 0.43		0.6006	2.51 ± 0.27		0.2180
**$F1_9$**	**CCR**	1.02 ± 0.02	0.2879	0.7595	8.11 ± 0.54	0.9771	**<0.0001**	3.17 ± 0.32	0.3940	**<0.0001**
	**IF**	1.21 ± 0.07	0.3804	0.5925	9.46 ± 0.57	0.2504	**0.0005**	2.86 ± 0.38	0.9625	**0.0181**

Supplementary Table 3. Changes in length of pre-reproductive, reproductive, and post-reproductive periods in offspring from 3, 5, 7, and 9-d old mothers ($F1_3$, $F1_5$, $F1_7$, and $F1_9$, respectively) under *ad libitum* (AL; 6 x 10^5 cells ml^{-1} *Tetraselmis suecica*), chronic caloric restriction (CCR; 6 x 10^4 cells ml^{-1} *T. suecica*, a 90% reduction in food relative to AL), or intermittent fasting (IF; alternate day AL and starvation) diets. Significant differences ($p < 0.05$) are shown in bold.

	Diet	Age at Max. Repro (d)	Diff than AL? (p)	Diff than $F1_3$? (p)	Age at Repro Senesc. (d)	Diff than AL? (p)	Diff than $F1_3$? (p)	Discont. Repro Period (%)	Diff than AL? (z, p)	Diff than $F1_3$? (z, p)
	**AL**	5.38 ± 0.14			10.97 ± 0.21			2.8		
**$F1_3$**	**CCR**	5.55 ± 0.18	0.697		13.45 ± 0.36	**<0.0001**		17.8	2.94, **0.0032**	
	**IF**	4.89 ± 0.14	**0.048**		13.89 ± 0.37	**<0.0001**		45.8	5.98, **<0.0001**	
	**AL**	4.49 ± 0.11		**0.0002**	9.84 ± 0.23		0.163	6.3		0.97, 0.333
**$F1_5$**	**CCR**	4.52 ± 0.16	0.989	**<0.0001**	11.46 ± 0.35	**0.0112**	**0.0016**	15.9	2.99, 0.083	0.09, 0.76
	**IF**	4.39 ± 0.09	0.909	0.1187	10.47 ± 0.44	0.5325	**<0.0001**	38.9	4.32, **<0.0001**	0.61, 0.780
	**AL**	5.20 ± 0.15		0.825	8.60 ± 0.27		**<0.0001**	4.3		0.47, 0.64
**$F1_7$**	**CCR**	5.26 ± 0.21	0.955	0.5173	10.99 ± 0.45	**<0.0001**	**<0.0001**	14.1	4.04, **0.044**	0.37, 0.54
	**IF**	4.52 ± 0.17	**0.004**	0.3041	13.90 ± 0.39	**<0.0001**	>0.9999	57.8	6.85, **<0.0001**	1.43, 0.154
	**AL**	4.23 ± 0.11		**<0.0001**	7.56 ± 0.42		**<0.0001**	7.8		1.23, 0.199
**$F1_9$**	**CCR**	4.21 ± 0.16	0.994	**<0.0001**	9.22 ± 0.53	**0.0085**	**<0.0001**	12.3	0.88, 0.381	0.81, 0.37
	**IF**	4.28 ± 0.12	0.977	**0.0342**	10.66 ± 0.56	**<0.0001**	**<0.0001**	31.5	3.33, **0.0009**	1.63, 0.103

Supplementary Table 4. Changes in ages of maximum reproduction and reproductive senescence and in the continuity of the reproductive period in offspring from 3, 5, 7, and 9-d old mothers ($F1_3$, $F1_5$, $F1_7$, and $F1_9$, respectively) under *ad libitum* (AL; 6 x 10^5 cells ml^{-1} *Tetraselmis suecica*), chronic caloric restriction (CCR; 6 x 10^4 cells ml^{-1} *T. suecica*, a 90% reduction in food relative to AL), or intermittent fasting (IF; alternate day AL and starvation) diets. Significant differences ($p < 0.05$) are shown in bold.
